# (*E*)-3-(3-Methyl­thio­phen-2-yl)-1-*p*-tolyl­prop-2-en-1-one. Corrigendum

**DOI:** 10.1107/S2414314621010774

**Published:** 2021-10-29

**Authors:** S. Naveen, Helmi Mohammed Al-Maqtari, Joazaizulfazli Jamalis, Hasnah Mohd Sirat, N. K. Lokanath, Muneer Abdoh

**Affiliations:** a Institution of Excellence, University of Mysore, Manasagangotri, Mysuru 570 006, India; b Faculty of Science, Universiti Teknologi Malaysia, 81310 UTM Skudai Johor, Malaysia; c Department of Studies in Physics, University of Mysore, Manasagangotri, Mysuru 570 006, India; d Department of Physics, Science College, An-Najah National University, PO Box 7, Nablus, West Bank, Palestinian Territories; University of Aberdeen, Scotland

## Abstract

In the paper by Naveen *et al.* [ *IUCrData* (2017), **2**, x170234], there was an error in the name of one of the authors.

## Abstract 2

Corrigendum to *IUCrData* (2017), **2**, x170234.

The name of the second author in the paper by Naveen *et al.* (2017 ▸) is incorrect and should be ‘Helmi Mohammed Al-Maqtari, as given above.
